# Purification of a peptide tagged protein via an affinity chromatographic process with underivatized silica

**DOI:** 10.1002/elsc.202100019

**Published:** 2021-06-04

**Authors:** Stefan Rauwolf, Tobias Steegmüller, Sebastian Patrick Schwaminger, Sonja Berensmeier

**Affiliations:** ^1^ Department of Mechanical Engineering Technical University of Munich Munich Germany

**Keywords:** affinity chromatography, amino acids, peptide tag, protein purification, silica

## Abstract

Silica is widely used for chromatography resins due to its high mechanical strength, column efficiency, easy manufacturing (i.e. controlled size and porosity), and low‐cost. Despite these positive attributes to silica, it is currently used as a backbone for chromatographic resins in biotechnological downstream processing. The aim of this study is to show how the octapeptide (RH)4 can be used as peptide tag for high‐purity protein purification on bare silica. The tag possesses a high affinity to deprotonated silanol groups because the tag's arginine groups interact with the surface via an ion pairing mechanism. A chromatographic workflow to purify GFP fused with (RH)4 could be implemented. Purities were determined by SDS‐PAGE and RP‐HPLC. The equilibrium binding capacity of the fusion protein GFP‐(RH)4 on silica is 450 mg/g and the dynamic binding capacity around 3 mg/mL. One‐step purification from clarified lysate achieved a purity of 93% and a recovery of 94%. Overloading the column enhances the purity to >95%. Static experiments with different buffers showed variability of the method making the system independent from buffer choice. Our designed peptide tag allows bare silica to be utilized in preparative chromatography for downstream bioprocessing; thus, providing a cost saving factor regarding expensive surface functionalization. Underivatized silica in combination with our (RH)4 peptide tag allows the purification of proteins, in all scales, without relying on complex resins.

**Abbreviations**
BC: big column
CV: column volume
DBC: dynamic binding capacity
EBC: equilibrium binding capacity
Egfp: enhanced green fluorescent protein
PB: phosphate buffer
SC: small column

## 1 INTRODUCTION

Downstream processing is currently the costliest aspect during protein purifications [1]. Especially the biotechnological production of pharmaceuticals (e.g. antibodies, enzymes), with necessary purities of more than 99%, require several processing steps [1, 2]. A frequent choice of method is the chromatographic separation of molecules. The chromatographic separation can be based upon several interactions [3]. Affinity chromatography, as one of the most selective separation methods, seems to be inevitable when trying to achieve maximum purities of the product [2]. Affinity chromatography relies on specific binding between two ligands [4]. Therefore, peptide tag systems such as Strep‐tag, poly(His)‐tag, Maltose‐binding protein, and so on. are often used to achieve a strong chemical interaction to the functionalized stationary phase [5, 6, 7]. The benefit of a tag system is the ability to bind the target protein specifically, through molecular engineering; therefore, achieving high purities after a single step. However, in most tag systems the stationary phase needs to be functionalized to fit the properties of desired chemical interaction [5, 7]. Functionalizations of the resin are often unstable and reduce the overall number of available binding sites. Reduced capacity and limited lifetime are reflected in the process costs [5].

Silica, an abundant resource on earth, is inexpensive and the most used material in liquid chromatography (LC), especially in reversed phase, normal phase, and hydrophilic LC [8, 9, 10]. It offers low counter pressure and the silanol groups on the surface can be easily functionalized with various surface modifications [10]. The silanol groups, negatively charged at pH >2–3, can undergo electrostatic interactions which are not favored in conservative silica applications [10, 11, 12]. However, there are only a few studies on protein purification on underivatized silica [13, 14]. Recent developments suggest that peptide tags enable the purification of proteins with bare silica, putting silica resins back in the focus for protein purification research [15, 16, 17, 18, 19, 20, 21].

In aqueous systems silica interacts mainly with the basic amino acids lysine and arginine via electrostatic interactions [22, 23, 24]. This was also shown by our group in a previous study for different buffer systems [25]. These findings led to the idea to use one of our peptide tags, which have been originally designed for bare magnetic nanoparticles: (RH)4 [26]. The (RH)4‐tag is a short peptide, consisting of four consecutive arginine‐histidine groups with a total of eight amino acids and can be used for immobilized metal affinity chromatography (IMAC) as well [26]. For this study, previous work on silica related tags was considered. However, these works have been done in static binding systems such as a batch method, involved larger peptide tags and were applied on a smaller scale [15–20, 27–29]. In this study the (RH)4‐tag system was implemented for protein purification in a conventional chromatographic workflow. The process shows to be applicable on a larger scale and due to its small size of eight amino acids, the influence on the POIs biological activity and structure are minimized [30].

A functional process using underivatized silica would be inexpensive and easily applicable for many proteins. This study demonstrates the capture of a target protein (i.e. eGFP), with the designed (RH)4 tag system, on an underivatized silica matrix. eGFP was used as model protein since its fluorescence at 488 nm makes it easy to detect [31]. The protein could be captured, and a fully dynamic chromatographic workflow was developed. With a single step chromatography ∼90–95% pure GFP‐(RH)4 is recovered. Silica in its nature has a huge specific surface area, e. g. the Davisil 643 used in this study has 300 m^2^/g. Therefore, by using it as a stationary phase, a high binding capacity could be observed. Effectiveness of the (RH)4 tag chromatographic workflow is shown in its high purities after only one‐step. Thus, an application‐ready process could be developed for purification of (RH)4‐tagged proteins on bare silica resins.

**PRACTICAL APPLICATION**

Underivatized silica is an abundant and cheap material with outstanding performance especially in analytical chromatographic processes and has yet to find its place in protein purification. Chromatography is one of the most important unit operations in protein purification scenarios. There is a need for new, affordable, and innovative methods in downstream processing of proteins. Our rationally designed (RH)4 peptide tag in combination with underivatized silica as a stationary phase, allows an easy and inexpensive affinity purification process of fusion proteins. In laboratory scale runs, the fully dynamical process is automated from equilibration over loading, elution, and column washing and completed within 1–2 h depending on the amount of lysate. Purities of >95% are achieved in a single step with a recovery of about 94%. In regards to column packing, Silica is easy to handle, thus, allowing proving this method useful for beginner to advanced chromatographers with potential for up‐scaling.

## 2 MATERIALS AND METHODS

### 2.1 Materials

All solvents and chemicals were of analytical grade. Buffers used for preparative and analytical chromatography were filtered (0.2 μm ∅) and degassed. The cloning of the GFP‐(RH)4 variant was published earlier by our group [26, 32]. The *E. coli* strain BL21DE was used and incubated at 37°C in baffled flasks, at 150 rpm until an OD600 of 0.7 was achieved. After induction with 1 mM IPTG, the protein expression was carried out at 16°C at 150 rpm. For chromatographic experiments a 10 × 100 mm Omnifit column (Kinesis, Germany) filled with Davisil 643 (Sigma. Germany) was used. The bed volume was set to 1.5 mL if not stated otherwise. The column was connected to an ÄKTApurifier (GE Healthcare, Germany).

### 2.2 Characterization of tag‐silica interaction

For binding experiments GFP‐(RH)4 was purified via IMAC as reported previously [26]. To achieve the cleavage, a 1:100 w/w ratio of a 1000 U TEV‐protease to protein were mixed. The mixture was placed in a 32 mm wide dialysis tube (Thermo Fisher Scientific Inc, USA, MWCO 10000). After the tube was locked it was incubated overnight in 2 L of a 50 mM Tris buffer (pH 8.0). Both intact GFP‐(RH)4 and a TEV‐cleaved GFP‐(RH)4 were injected into a silica column using 50 mM Tris pH 8.0.

For the dynamic binding capacity (DBC) a concentration (c_p_) of 1 mg/mL GFP‐(RH)4 was first measured via the systems bypass, to gather the max reference absorbance. The GFP‐(RH)4 was then directed through a silica column with a column volume (CV) of 1.5 mL until a breakthrough curve could be observed. The DBC, at 10% of the obtained max mAU value, was then calculated with equation 1.

(1) $$DBC_{10}=\frac{c_{p}*Q*t_{10}}{CV}$$

With *Q* being the volumetric flowrate and t_10_ the time passed until the 10% breakthrough curve occurred.

For the static equilibrium binding capacity (EBC), 1 g/L silica particles were supplemented with set dilutions of GFP‐(RH)4 (3, 2, 1.5, 1, 0.75, 0.5, 0.2, 0.1 g/L; purity >95%). A supernatant analysis via UV/vis and as an orthogonal analysis a particle‐BCA were performed to assess the total amount of protein bound to the silica particles and calculate the static binding capacity, as well as the *K*_d_.

Therefore, the collected pellets were washed three times with buffer. After washing, the samples were transferred to a filter 96‐well plate on top of a regular 96‐well plate. The assembled plates were centrifuged at 3000 × *g* for 10 min to remove remaining liquid. The BCA assay was then carried out with the Pierce BCA protein assay kit (Thermo Fisher Scientific Inc., USA). After incubation, the stacked plates were centrifuged at 3000 × *g* for 30 min until the BCA reagent passed through the filter into the 96‐well plate below. The absorbance at 562 nm was measured via an Infinite M200 microplate reader (Tecan Deutschland, Germany). All samples were analyzed in analytical and technical triplicates

### 2.3 Chromatographic purification of fusion protein

Cell pellets were resuspended in 50 mM Tris‐HCl pH 8.0 and supplemented with protease inhibitor (Roche, Switzerland), EDTA (Carl Roth, Germany) and DNAse I (AppliChem, Germany). Cell lysis was performed via French press (Julabo GmbH, Germany) at 1.8 kbar. The lysate was centrifuged at 20,000 rpm for 50 min at 4°C to collect the soluble proteins of interest and hold on ice during the whole process. The column was equilibrated for four CV with 50 mM Tris‐HCl, 5% Glycerol, pH 8.0 at a flowrate of 1 mL/min. The equilibration step was followed by loading of the cleared lysate onto the column. Once the lysate was loaded on the column, it was washed with four CV equilibration buffer. As soon as the UV signal for 280 nm decreased back to the baseline, the elution process was started. For elution 50 mM Tris‐HCl buffer was supplemented with 0.5 M l‐lysine, pH 8.0.

### 2.4 Buffer experiments

For the buffer experiments three different buffer system were prepared, 50 mM MOPS pH 8.0, 50 mM Phosphate pH 8.0, and 50 mM Tris pH 8.0. All samples were prepared in technical triplicates. 0.1 g of silica were added to 1 mL of the respective buffer and supplemented with 1 mL of the respective cleared lysate. The mixture was incubated overnight at 16°C at 1200 rpm. After incubation, the tubes were centrifuged at 16,000 × *g* for 5 min and the supernatant was removed. The washing step was repeated twice. After washing, 0.5 M l‐lysine dissolved in the respective buffer system was added to the tubes. The mixture was incubated for 1 h at 16°C. After incubation, the tubes were centrifuged, and the supernatant was collected and analyzed via BCA and HPLC.

### 2.5 Protein analysis

The concentration and purity were determined by UV/Vis, SDS‐PAGE, and RP‐HPLC.

The amount of protein in solution was determined via UV/Vis spectroscopy and the measured values were transformed via Beer‐Lambert law (Equation 2).

(2) $$A_{489}=\;\varepsilon *d*c$$

With *A* being the measured absorbance value at 489 nm, the extinction coefficient of eGFP at ε_489nm_ = 56,000 M^−1^ cm^−1^ [31], *d* the path length, and *c* the molar concentration.

For SDS‐PAGE the samples were mixed with a SDS loading buffer (containing 10 mM DTT), heated for 5 min at 95°C, and loaded onto a 12% polyacrylamide gel unless stated otherwise. The gel was scanned with the high‐resolution scanner Amersham Typhoon NIR Plus (GE Healthcare Europe GmbH, Germany), and the densitometric analysis was performed with its analysis software Image Quant TL.

For RP‐HPLC analysis 8 μL of approximately 0.5 g/L protein sample was loaded onto a C4 column (Aeris, 3.6 μm, Widepore, 150 × 2.1 mm). The samples were analyzed three times and the following buffers were used: buffer A ‐ ddH_2_O with 20 mM TFA; buffer B ‐ 100% acetonitrile with 20 mM TFA. The gradient ran from 40% to 60% B in ten CV followed by three CV at 100% B and an equilibration step of five CV at 40% B. For evaluation, all peaks at 233 nm were integrated and the purity was calculated using the ratio of the eGFP peak to the total protein peak area subtracting the buffer peaks from the total peak area.

## 3 RESULTS AND DISCUSSION

### 3.1 Characterization of the tag‐silica interaction

**FIGURE 1:**
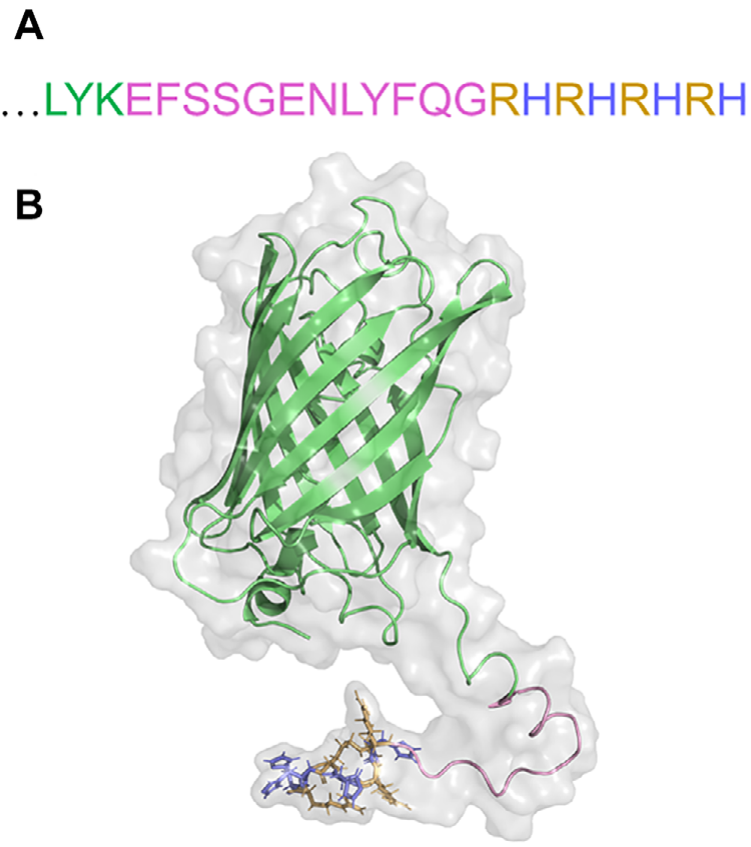
(A) Sequence of the C‐terminal attachment of (RH)4 to GFP and (B) 3‐dimensional structure of GFP‐(RH)4 via Robetta simulation. The GFP sequence (green) ends with Leu‐Tyr‐Lys followed by an SSG‐linker, a TEV‐protease site (pink) and ends with the arginine (yellow)‐histidine (blue) tag

For a short octapeptide such as (RH)4 it is crucial for the tag to be separated from the protein and able to interact with the silica surface, without forcing the protein to the surface of the highly negative charged silica. This is achieved by inclusion of a short linker sequence (SSG) between protein and tag as well as a protease restriction site (TEV protease) as indicated in Figure 1A The online deep learning protein structure prediction service Robetta provided by the Baker Lab at the University of Washington was used, to get a preliminary understanding of the general structure of the fusion protein (see Figure 1B) [33, 34]. For better understandability, the eGFP is marked in green, the linker + TEV site in pink, and the (RH)4‐tag in yellow and blue for arginine and histidine, respectively. The structure prediction shows that the peptide tag is super exposed from the protein and thus has no steric hindrance for binding.

The design of the tag allows for it to be positively charged while the overall protein remains negatively charged at alkaline pH. At acidic pH, the whole protein will be positively charged, since GFP‐(RH)4 shows a theoretical pI of 6.11 (ExPASy); thus, binding to the negatively charged silica matrix, alongside many other proteins [13, 35–37]. Therefore, a basic pH is preferable since the protein will be repelled by the negative silanol groups, whereas the positively charged tag is still able to bind to the stationary phase. Keeping the stability of the protein and silica in mind, a pH of 8.0 was chosen to satisfy these parameters. A pH between 7.5 and 8.5 was also used by other groups for silica binding peptides such as Car9 or the Si‐Tag [15, 17, 29].

**FIGURE 2:**
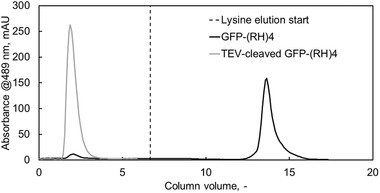
Chromatogram of GFP‐(RH)4 (black) and TEV‐cleaved GFP‐(RH)4 (grey) with 50 mM Tris pH 8.0 as running buffer. TEV‐cleaved GFP‐(RH)4 eluted after roughly one column volume indicating no binding to the silica stationary phase. The intact GFP‐(RH)4 eluted only after switching to the eluting buffer (dashed black line) supplemented with 0.5 M l‐lysine

To proof experimentally that the (RH)4 peptide is responsible for the binding, intact GFP‐(RH)4 and TEV protease cleaved GFP‐(RH4) were compared for their binding ability under the same conditions. As shown in Figure 2 the intact GFP‐(RH)4 bound to the column and did not elute until L‐lysine was added. As displayed in Figure 2 elution took around five CV to start indicating potential in optimizing the elution step by testing different lysine concentrations or other eluting agents such as arginine [27]. The cleaved eGFP did not bind to the silica stationary phase and started eluting after 1 CV indicating that the (RH)4 is responsible for protein binding.

For elution l‐lysine was chosen because, as previously stated, NaCl is not suitable to achieve good elution and amino acids such as lysine and arginine showed a preferable result [19, 28, 29, 38]. Using these amino acids has the additional advantage of stabilizing eluted proteins [39, 40]. Eluting with salts such as NaCl and MgCl_2_ (1 M) was not possible and seems to be bothersome even at high concentrations (1–5 M) [15, 17, 41]. Lysine's and arginine's competitive elution effects work at moderate concentrations of around 0.5 M but were also reported to elute at lower concentration (0.1 M) [20]. Concluding previous studies on amino acids [22, 25], peptides [42], and protein interactions with silica [14, 43], electrostatic interactions play the main role in binding. In case of (RH)4 the four positively charged arginine groups in the tag interact with the negatively charged silanol groups on the silica surface. Although, silica showed to be a weak ion exchanger [35], the resistance to salt elution in our study would suggest otherwise. In classical ion exchange chromatography NaCl gradients up to 400 mM trigger protein elution and regeneration of columns is performed with 1 M NaCl [44]. Elution of the Arg‐tag, consisting of five to six arginine residues, was also shown to be possible with the classical setup [6, 45]. Considering the resistance of different silica binding peptides to salt elution from bare silica, the binding mechanism does not seem to be a classical ion exchange, but rather ion pairing. This binding mechanism was already suggested in a previous study where binding of cationic peptides to silica showed no change in the distribution of Na^+^ ions in solution and binding occurred even at low initial peptide concentration thresholds. [42].

The (RH)4‐tag with its eight amino acids is a short peptide‐tag capable of enhancing the thermostability of fused proteins and due to its rational design allows binding to multiple surfaces such as silica, magnetic nanoparticles and immobilized metal ions in IMAC [26]. IMAC is still one of the most used chromatographic techniques for protein purification [7, 46], making the (RH)4‐tag an affinity tag for two chromatographic systems.

### 3.2 Binding capacities of silica

**FIGURE 3:**
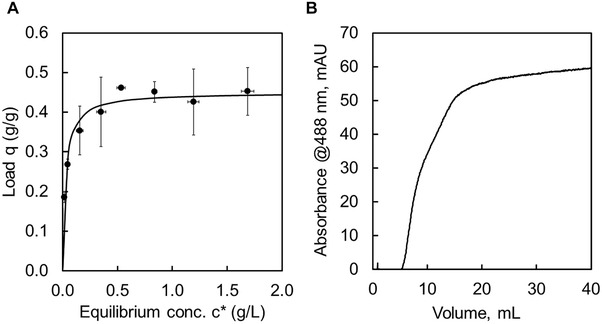
(A) Equilibrium binding capacity (EBC) at 50 mM Tris pH 8.0 and 1 g/L silica. Adsorption isotherm after Langmuir model. (B) Breakthrough curve of dynamic binding capacity (DBC_10_) for 1 g/L GFP‐(RH)4 with 50 mM Tris pH 8.0 on silica with at a flowrate of 1 mL/min (column volume 1.5 mL)

Purification of a protein with a silica affinity tag was previously optimized for GFP regarding silica type, buffers, and pH [20, 29, 41]. Therefore, Davisil 643 silica particles were chosen and Tris buffer at pH 8.0. The silica particles possess a narrow size distribution of 35–70 μm enabling homogenous packing in the column and promises good chromatographic resolution. Since Davisil 643 is a porous silica with a large specific surface area of 300 m^2^/g, pores of 15 nm diameter, and a pore volume of 1.15 cm^3^/g (given by manufacturer), a high binding capacity for relatively small proteins such as eGFP is expected and an important property for chromatographic materials. It should be noted, that for larger proteins such as antibodies a silica with larger pores could be more suitable due to pore‐diffusion issues [47]. The equilibrium binding capacity (EBC, Figure 3A) and the dynamic binding capacity (DBC_10_, Figure 3B) for GFP‐(RH)4 on silica were determined in this study. The resulting adsorption isotherm of EBC experiments can be described by the Langmuir model with a dissociation constant K_D_ = 0.02 g/L (0.7 μM) and maximum load q_max_ = 450 mg/g. The *K*_D_ is in the same order of magnitude as for our magnetic nanoparticles [26], and also comparable to the equivalent small Car9 peptide [15]. The maximum load is higher or in the same order of magnitude as other protein loadings on silica [35, 48, 49].

In contrast to the EBC, which shows the maximum load of a protein to a material, the dynamic binding capacity (DBC_10_) takes the chromatographic process parameters into account. Thus, the DBC_10_ yields the binding capacity under operating conditions. The DBC_10_ for the 1.5 mL column was performed at 1 mL/min and a protein concentration of *c*_P _= 1 g/L. The dead volume of 1.6 mL was previously determined and includes the column and wires. With the breakthrough curve (Figure 3B) the DBC_10_ calculates to ∼3 mg/mL, and is comparable to other affinity tag systems [5, 15]. However, due to silica's nature of high surface area and lack of functionalizations, a higher capacity even under operating conditions is expected. Improvements on the binding capacity may be achieved by varying the process parameters such as pH or ionic strength of the buffers.

### 3.3 Purification process from clarified lysate

**FIGURE 4:**
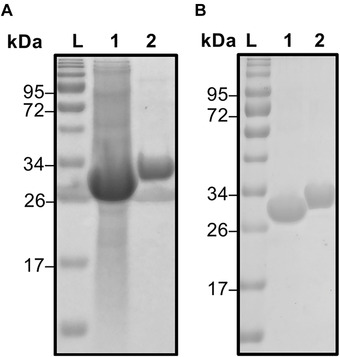
(A) SDS‐PAGE of GFP‐(RH)4 purification out of lysate with lysate (1) and elution fraction (2). (B) SDS‐PAGE of TEV‐protease digestion of GFP‐(RH)4 with digested GFP‐(RH)4 (1) and an intact GFP‐(RH)4 (2). Protein standard ladder (L) for comparison in both gels

For the purification process the chromatographic column was set to a CV of 1.5 mL and operated at 1 mL/min. Five hundred microliter lysate were injected via a sample loading loop. With these parameters, a purity of 90–93% by HPLC and SDS‐PAGE evaluation (see Figure 4) with a recovery of about 94% has been achieved. The protein purity is comparable to purities gained with other peptide tags such as SB7 and Car9 [27, 29]. However, the recovery in our system is higher compared to both systems with ∼65% and 75–90%, respectively. Loss of protein occurs due to tag degradation and during the ultrafiltration process. Considering that GFP‐(RH)4 is already overexpressed and abundant (SDS‐PAGE ∼60%) in the lysate the selective binding of (RH)4 enables a one‐step purification of fusion proteins out of lysates in a classical chromatographic workflow. Previous studies on silica binding tags either bound in static systems or in very small scaled spin columns with 600 μL working volume [15, 19, 20, 29]. In this study a fully working chromatographic workflow was implemented, which allows real time monitoring of the loading, washing, and elution step. The degradation of the tag can be a result due to its super exposed nature or problems in the sequence which can lead to degradation by proteases [15, 50]. A degradation of the tag (26 kDa eGFP, 29 kDa GFP‐(RH)4) could be possible and would explain the additional band which can be seen in Figure 3A in the lysate (lane 2) and the purified fraction (lane 3). This was confirmed by a TEV‐protease digestion of GFP‐(RH)4 which was compared to an untagged eGFP standard (Figure 4B). Subsequently, EDTA and 5% glycerol were used, as additional additives to support the protein's stability [51]. Impurities in the process are caused by non‐specific binding of proteins [14, 29]. The main contaminant around 43 kDa most likely seems to be an RNA‐binding protein with natural high affinity to silica [29, 52].

### 3.4 Up‐scaling and optimization of process parameters

**FIGURE 5:**
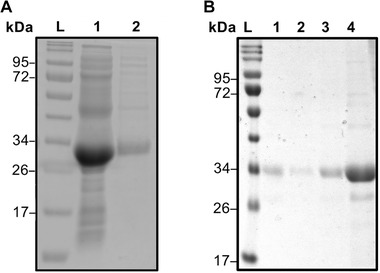
(A) SDS‐PAGE with lysate (1) and elution fraction (2) of XK16 column with a column volume of 75 mL. The column was loaded with 50 mL lysate. (B) SDS‐PAGE analysis of elution fractions from different columns and injection volumes. A 1 mL column with injections of 50 μL (1) and 500 μL (2) and a 2 mL column with injections of 50 μL (3) and 500 μL (4) of 2 g/L GFP‐(RH)4. Protein standard ladder (L) for comparison in both gels

The potential of process‐up‐scaling was investigated. Therefore, a XK16 column was prepared (GE Healthcare, Germany) with a column volume of 75 mL. The column was loaded with 50 mL lysate (with a eGFP concentration of 2 g/L). The column, which was only partially loaded, (indicated by a green color change of the stationary phase) showed a purity of >80% after elution, also indicated by multiple protein bands in the SDS‐PAGE (Figure 5A, lane 2). An explanation for the reduced purity could be that many binding sites were left open for other lysate proteins to bind to silica, due to only partially loading the column. However, it was possible to up‐scale the process (i.e. for preparative capture steps), regarding polishing there is potential for optimization. Theoretically, the specific binding of the (RH)4‐tag should be stronger than unspecific binding of other proteins. Therefore, different volumes (50 and 500 μL) of the same lysate were loaded on a small column with a column volume of 1 mL (SC) and a bigger column (BC) with a volume of 2 mL. The size of the column and the amount of protein loaded consequently impacted the purity. For the small column with a CV of 1 mL even 50 μL of the lysate were enough to have to achieve a purity >95% (Figure 5B, lane 1+2). The BC with a CV of 2 mL showed a purity of roughly 92% when injecting 500 μL lysate (Figure 5B, lane 3). The BC showed a purity over 95% upon overloading with the lysate (Figure 5B, lane 4) indicating that the competitive effect of the (RH)4‐tag enhances purity. The competitive effect of the target protein could not occur on the XK16 column; thus, resulting in a lower purity indicating the importance of the equilibrium on the column. These results show the limitation and application potential of the chromatographic method and also the silica‐peptide system itself: Overloading the silica, to get high purities, lead to loss in protein recovery; high protein recovery leads to decrease in purity. However, the loss in protein recovery for high purity can be minimized due to real‐time measurement of eGFP at 488 nm. For other proteins this is more challenging as they do not have a unique absorption wavelength and the amount of lysate for loading must be calculated.

### 3.5 Influence of the buffer system on the process

**FIGURE 6:**
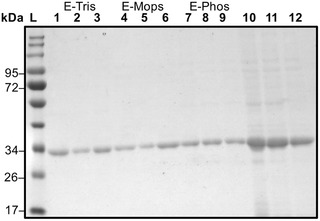
SDS‐PAGE of elution fractions from static buffer experiments of 50 mM Tris (E‐Tris: 1–3), MOPS (E‐MOPS: 4–6), and phosphate (E‐Phos: 7–9) at pH 8.0. Lysate in respective buffers for illustration (10‐12). Protein standard ladder (L) for comparison

As previously shown, the buffer can greatly influence the interaction of biomolecules with silica [25]. Until now, studies on silica affine peptide tags used mainly Tris buffer for the binding and purification process. However, due to the binding mechanism other buffer system should work as well. Consequently, the influence of different buffer systems on the purification system was investigated. Three buffers were chosen which can buffer in the region of pH 8.0: Tris (bearing a positive charge), phosphate (PB, bearing a negative charge), and MOPS (bearing a positive and a negative charge). Figure 6 suggests that the GFP‐(RH)4 purity is not influenced by the buffer species and therefore buffer charge. Purities >90% were achieved for every buffer in the static system (Figure 6).

In classic ion exchange the buffer system impacts the process due to electrostatic interactions between charged buffer species and stationary phase [53]. However, buffer charge seems not to significantly influence our process, providing further evidence for the binding mechanism not being ion exchange but a more affinity like ion pairing mechanism which allows the (RH)4‐tag system to be used with a variety of buffer systems.

## 4 CONCLUDING REMARKS

Chromatographic processes remain the most important unit operation for achieving high purities in protein purification. For this purpose, Underivatized silica in combination with the (RH)4‐tag is a promising method. A conventional chromatographic process for the purification of GFP‐(RH)4 was implemented, with resulting purities of >90% and a recovery of >94%. The process can be easily up‐scaled, considering that the column needs to be loaded completely with the protein of interest. The enhancing effect of overloading the column and improving the purity of the protein of interest to >95% could be shown. Our system proved to be independent from the buffer species, leading to a more flexible use of this method. This process is immensely versatile, in that it can be both up‐ and downscaled for industrial or laboratory use; respectively enabling a widespread use for high‐purity (RH)4 tagged proteins among a wide range of buffer systems. The most promising application of this system would be as capture or polishing step in combination with another chromatographic system such as IMAC or ion exchange chromatography, which both are frequently used in protein chromatography. We are currently investigating the up‐scaling and the transferability of the (RH)4 tag system to other proteins and enzymes, which are not as overexpressed and abundant in the lysate as eGFP, for purification and immobilization.

## CONFLICT OF INTEREST

The authors have declared no conflicts of interest.

## Data Availability

The data that support the findings of this study are available from the corresponding author upon reasonable request.
